# BCL2L10 Is Overexpressed in Melanoma Downstream of STAT3 and Promotes Cisplatin and ABT-737 Resistance

**DOI:** 10.3390/cancers13010078

**Published:** 2020-12-30

**Authors:** María Josefina Quezada, María Elisa Picco, María Belén Villanueva, María Victoria Castro, Gastón Barbero, Natalia Brenda Fernández, Edith Illescas, Pablo Lopez-Bergami

**Affiliations:** 1 Centro de Estudios Biomédicos, Básicos, Aplicados y Desarrollo (CEBBAD), Universidad Maimónides, C1405BCK Buenos Aires, Argentina; quezada.josefina@maimonides.edu (M.J.Q.); villanueva.belen@maimonides.edu (M.B.V.); castro.victoria@maimonides.edu (M.V.C.); barbero.gaston@maimonides.edu (G.B.); illescas.edith@maimonides.edu (E.I.); 2 Consejo Nacional de Investigaciones Científicas y Técnicas (CONICET), C1405BCK Buenos Aires, Argentina; 3 Instituto de Medicina y Biología Experimental (IBYME), CONICET, C1428ADN Buenos Aires, Argentina; mariaelisapicco@gmail.com (M.E.P.); nfernandez@cerebro.fbmc.fcen.uba.ar (N.B.F.)

**Keywords:** BCL2L10, STAT3, melanoma, cytotoxicity, survival, ABT-737, Bcl-2 family, ML258

## Abstract

**Simple Summary:**

BCL2L10 is the sixth and less studied protein from the group of Bcl-2 anti-apoptotic proteins. These proteins are important therapeutic targets since they convey resistance to anticancer regimens. We describe here for the first time the role of BCL2L10 in melanoma. We found that BCL2L10 is abundantly and frequently expressed both in melanoma cell lines and tumor samples. This increased expression is due to the activity of the transcription factor STAT3 that positively regulate BCL2L10 transcription. We describe that Bcl2l10 is a pro-survival factor in melanoma, being able to protect cells from the cytotoxic effect of different drugs, including cisplatin, dacarbazine, and ABT-737. BCL2L10 also inhibited the cell death upon combination treatments of PLX-4032, a BRAF inhibitor, with ABT-737 or cisplatin. In summary, we determined that BCL2L10 is expressed in melanoma and contributes to cell survival. Hence, targeting BCL2L10 may enhance the clinical efficacy of other therapies for malignant melanoma.

**Abstract:**

The anti-apoptotic proteins from the Bcl-2 family are important therapeutic targets since they convey resistance to anticancer regimens. Despite the suspected functional redundancy among the six proteins of this subfamily, both basic studies and therapeutic approaches have focused mainly on BCL2, Bcl-xL, and MCL1. The role of BCL2L10, another member of this group, has been poorly studied in cancer and never has been in melanoma. We describe here that BCL2L10 is abundantly and frequently expressed both in melanoma cell lines and tumor samples. We established that BCL2L10 expression is driven by STAT3-mediated transcription, and by using reporter assays, site-directed mutagenesis, and ChIP analysis, we identified the functional STAT3 responsive elements in the BCL2L10 promoter. BCL2L10 is a pro-survival factor in melanoma since its expression reduced the cytotoxic effects of cisplatin, dacarbazine, and ABT-737 (a BCL2, Bcl-xL, and Bcl-w inhibitor). Meanwhile, both genetic and pharmacological inhibition of BCL2L10 sensitized melanoma cells to cisplatin and ABT-737. Finally, BCL2L10 inhibited the cell death upon combination treatments of PLX-4032, a BRAF inhibitor, with ABT-737 or cisplatin. In summary, we determined that BCL2L10 is expressed in melanoma and contributes to cell survival. Hence, targeting BCL2L10 may enhance the clinical efficacy of other therapies for malignant melanoma.

## 1. Introduction

Melanoma is the fifth and sixth most common cancer for men and women, respectively, and thus represents a major public health problem. In addition to its high incidence, melanoma is one of the most aggressive tumor types with a 5-year survival rate of around 20% [1]. At the molecular level, melanoma is characterized by the highly prevalent BRAF^V600E^ mutation that renders the MAPK/ERK pathway constitutively active and is critical for melanoma progression. Moreover, many other signaling pathways, such as PI3K/Akt, PKC, STAT3, Wnt, and Eph/ephrin, are also constitutively activated [2,3,4,5]. Targeted therapies toward BRAF and MEK and immunotherapy have shown promise in the management of this cancer, and currently, there are several single or combination therapies approved for first-line treatment of metastatic or unresectable disease [6]. However, melanoma remains difficult to treat due to innate and acquired resistance to these therapies. Sadly, this has been a recurrent problem in melanoma since the massive failure of standard chemotherapy nearly 50 years ago [7].

Melanoma is intrinsically resistant to diverse cytotoxic insults, such as DNA damage (e.g., by irradiation, alkylation, methylation or crosslinking), microtubule destabilization or topoisomerase inhibition. One of the underlying mechanisms is a profound dysregulation of cell death pathways in part due to the aberrant expression of proteins of the Bcl-2 family that regulates the intrinsic or mitochondrial apoptotic pathway [8]. There are at least 20 Bcl-2-related proteins that are categorized into one of the three subfamilies; anti-apoptotic proteins (BCL2, Bcl-xL/BCL2L1, Bcl-w/BCL2L2, MCL1, Bfl-1/BCL2A1, and BCL2L10), pro-apoptotic BH3-only proteins (BAD, BID, BIK, Bim/BCL2L11, BMF, HRK, NOXA, PUMA/BBC3, etc.) and pro-apoptotic pore-formers or executioners (BAX, BAK1, BOK). Proteins from the first two groups compete to influence the executioners that, if activated, form pores in the outer mitochondrial membrane and thus trigger mitochondrial outer membrane permeability (MOMP) and apoptosis [9,10]. Given its important role in cancer progression, anti-apoptotic members of the Bcl-2 family have been studied as therapeutic targets in cancer, first by using antisense oligonucleotides against BCL2 and Bcl-xL, and more recently by using the small molecule compounds called BH3-mimetics since they emulate the function of the BH3-only proteins. Currently, several BH3-mimetics are being evaluated as single agents or combined with other compounds in clinical trials for a wide number of tumor types, including melanoma [11].

BCL2L10 (also known as Bcl-b, NrH, Diva, or Boo) is the most recently identified and least studied protein of the Bcl-2 anti-apoptotic subfamily, and its function is only partially understood [12,13]. BCL2L10 has been classified into the anti-apoptotic group of Bcl-2 proteins since it contains all four BH domains, a distinctive feature that is missing in the other groups. Nevertheless, both pro-apoptotic [14,15,16,17,18] and anti-apoptotic [19,20,21,22] activities of BCL2L10 have been described. BCL2L10 was also found to have functions not related to apoptosis. In ovarian cancer and hepatocellular carcinoma, BCL2L10 acts as a tumor suppressor by negatively regulating cell proliferation [15,23]. Along this line, the BCL2L10 promoter was found to be aberrantly methylated in gastric cancer [14,18,24], hepatocellular carcinoma [17], and acute myeloid leukemia [25]. The ensuing inhibition of BCL2L10 expression correlated with a decreased overall survival and disease-free survival of hepatocellular carcinoma [17] and gastric cancer [26] patients. On the other side, BCL2L10 was found to be overexpressed in breast, prostate, colorectal, and lung cancers as well as in multiple myeloma [27,28,29,30]. In many of these tumor types, the elevated BCL2L10 expression was correlated with poor prognosis [27,28,29,30]. The reasons for these discrepancies in BCL2L10′s role in cancer have not been elucidated.

This study aimed to investigate the expression and function of BCL2L10 in melanoma since they have not been studied to the present. We found that BCL2L10 is expressed in both melanoma cell lines and patients and that its transcription is regulated by STAT3. In addition, we determined that BCL2L10 plays a pro-survival role in melanoma, protecting the melanoma cells from different cytotoxic insults.

## 2. Results

### 2.1. BCL2L10 Expression in Melanoma

**Figure 1:**
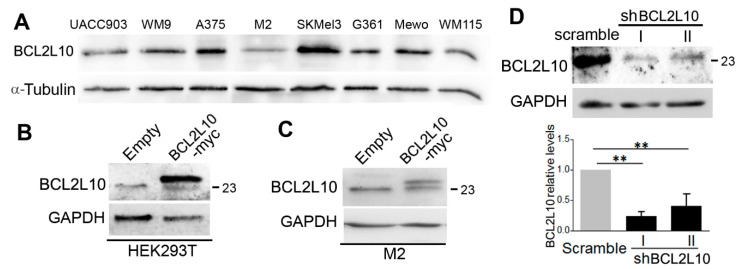
BCL2L10 expression in human melanoma cell lines and validation of BCL2L10 overexpression and silencing. (**A**) Protein extracts from the indicated melanoma cell lines were probed by Western blot with a 1:1000 dilution of the BCL2L10 antibody #3869. α-tubulin was used as the loading control. (**B**) Validation of the BCL2L10 antibody in HEK293 transfected with BCL2L10-myc. Protein extracts from HEK293T cells transfected with BCL2L10-myc or empty plasmid were assayed by Western blot using the anti-BCL2L10 PA5-22190 antibody (1:1000 dilution). (**C**) Validation of the BCL2L10 antibody in M2 cells stably transfected with BCL2L10-myc. Protein extracts from M2-empty and M2-BCL2L10-myc cells were assayed by Western blot using the PA5-22190 antibody (1:1000 dilution). (**D**) Silencing of BCL2L10 in A375 cells. Protein extracts from A375 cells transduced with two shRNA for BCL2L10 (I and II) or a scramble shRNA were analyzed for BCL2L10 expression by Western blot using the PA5-22190 antibody (1:1000 dilution). Bar graphs show the mean ± SD (from three independent experiments) of BCL2L10 levels normalized to the loading control and expressed as the fold change relative to scramble cells. The statistical analysis is described in Methods. **: *p* < 0.01, *n* = 3. α-tubulin was used as a loading control in panel A and GAPDH in panels B-D. The blots displayed in all panels are representative of three independent experiments. The 23 kDa marker is indicated in panels (**B**–**D**). Whole Western blot figures are provided in Supplemental Materials Figure S1.

We begin our study by determining the expression of BCL2L10 in a panel of eight melanoma cell lines by Western blot. Antibody #3869 detected the expression of BCL2L10 in all melanoma cell lines analyzed (Figure 1A).

Since the high frequency of BCL2L10 expression in our cell line collection may be due to a selection process inherent in creating tumor-derived cell lines or the conditions of “in vitro” cell culture, we sought to analyze BCL2L10 expression in melanoma specimens. However, this antibody did not result suitable this technique. Since BCL2L10 has been little studied, the detection of BCL2L10 in human tissues by immunohistochemistry (IHC) has been hindered by the lack of validated tools. The anti-BCL2L10 antibody PA5-22190 is one of the few commercial antibodies recommended for IHC, but to date, it has not been utilized in any scientific publication. Hence, to study BCL2L10 expression in melanoma patients, we first set to validate this antibody by Western blot and IHC analyses. An expression vector encoding human myc-tagged BCL2L10 was introduced into HEK293T cells, and BCL2L10 expression was evaluated in lysates from transfected cells. The PA5-22190 antibody detected a strong band corresponding to exogenous BCL2L10 at 24 kDa in the BCL2L10-myc transfected lysates, but not in lysates from cells transfected with empty plasmid (Figure 1B). Likewise, the antibody revealed a weaker band of about 23 kDa in both protein extracts, which corresponds with the expected molecular weight of the endogenous protein. The antibody detected the same two bands (of similar intensity in this case) in M2 melanoma cells stably transfected with BCL2L10-myc (Figure 1C). Importantly, no additional bands were detected, and the recognition pattern was similar to that of the BCL2L10 antibody #3869 (Supplementary Materials Figure S2). Expression of other Bcl-2 protein family members and that of other proteins implicated in apoptosis was not affected by BCL2L10 overexpression (Supplementary Materials Figure S3). To confirm the identity of the presumed endogenous BCL2L10 protein band, we transduced A375 melanoma cells with retroviral particles encoding two short-hairpin RNAs (shRNAs) specific for BCL2L10 or a scramble sequence (control shRNA), and the stable cell lines A375-shBCL2L10 I and II and A375-scramble were established. Western blot experiments revealed that the 23 kDa band is efficiently silenced in both A375-shBCL2L10 cell lines (Figure 1D). These results indicate that the PA5-22190 antibody is specific for BCL2L10.

**Figure 2:**
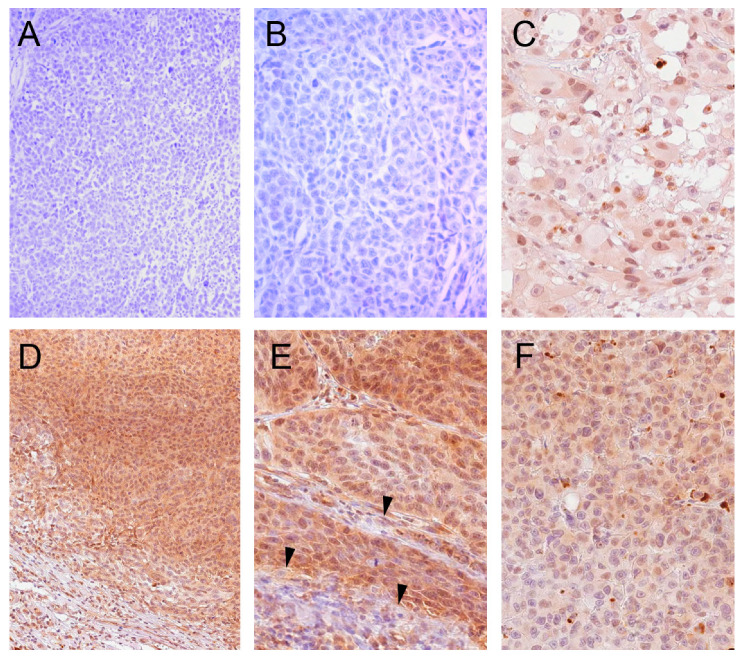
BCL2L10 is highly expressed in melanoma tumor samples. Representative images of immunohistochemistry staining for BCL2L10 using the PA5-22190 antibody (1:300 dilution) with hematoxylin counterstaining. (**A**,**B**) Representative images from a sample that scored negative for BCL2L10 staining (**A**, 100× magnification, **B**, 200× magnification). (**C**) Sample showing predominantly nuclear BCL2L10 staining (200× magnification). (**D**,**E**) Representative images from a sample showing nucleocytoplasmic BCL2L10 staining (**D**, 100× magnification, **E**, 200× magnification). BCL2L10 staining is not observed in the adjacent stroma (at the bottom on panel D) and in the septal stroma (arrows in panel E). (**F**) Sample showing predominantly cytoplasmic BCL2L10 staining (200× magnification).

To establish the conditions for IHC analysis of melanoma samples using the PA5-22190 antibody, we determined the antigen recovery method, antibody dilution, and other testing conditions (see Methods) using both positive (liver) and negative (placenta) control tissues that were selected based on BCL2L10 mRNA expression data from the Human Protein Atlas (www.proteinatlas.org). Then, we assayed samples from 20 melanoma patients from both primary and metastatic sites by IHC. Tissues were graded as strongly positive (+++), moderately positive (++), weakly positive (+), or negative (−). Robust expression of BCL2L10 was found in 90% of samples (scores ++ and +++), whereas the remaining 10% were negative (Figure 2 and Supplementary Materials Table S1).

BCL2L10 presented a heterogeneous pattern of immunoreactivity since both cytoplasmic and nuclear staining was observed both between- and within-samples. BCL2L10 staining was observed exclusively in the tumor tissue, whereas the immunoreactivity was not apparent in the adjacent noncancerous stroma (Figure 2D,E). These results demonstrate that BCL2L10 protein is frequently and strongly expressed in melanoma.

### 2.2. STAT3 Regulates BCL2L10 Expression

**Figure 3:**
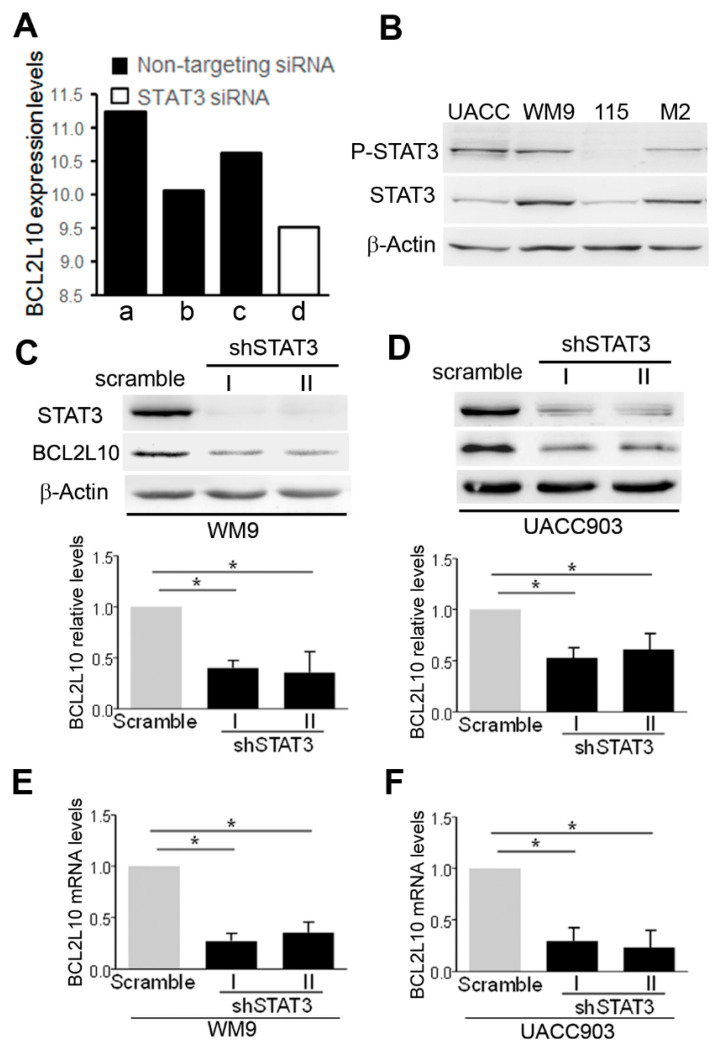
Silencing of STAT3-reduced BCL2L10 expression. (**A**) Regulation of BCL2L10 by STAT3 in dataset GSE31534. Analysis of dataset GSE31534 showed that a STAT3 siRNA (column d, GSM782740) reduced BCL2L10 mRNA levels in A375 melanoma cells compared to non-targeting siRNA (columns a to c: GSM782733, GSM782734, and GSM782735, respectively). (**B**) Phosphorylation of STAT3 at Tyr^705^ in melanoma cell lines. Protein extracts from UACC903 (UACC), WM9, WM115 (115) and M2 were probed with the indicated antibodies. β-actin was used as a loading control. The blots displayed are representative of three independent experiments. (**C**,**D**) Silencing of STAT3 in WM9 (**C**) and UACC903 (**D**) cells reduced BCL2L10 protein expression. UACC903 and WM9 cells were transduced with retrovirus encoding two STAT3 shRNA (I and II) and a scramble sequence as a control. Protein extracts were probed with antibodies to STAT3, BCL2L10 and β-actin as a loading control. The blots displayed are representative of three independent experiments. Bar graphs show the mean ± SD (from three independent experiments) of BCL2L10 levels normalized to the loading control and expressed as the fold change relative to scramble cells. *: *p* < 0.05, *n* = 3. (**E**,**F**) STAT3 silencing decreased BCL2L10 mRNA levels in WM9 (**E**) and UACC903 (**F**). Relative levels of BCL2L10 mRNA were determined by real-time PCR. mRNA levels, normalized to internal RNPII levels and expressed as relative to control cells. The mean ± SD from three independent experiments is shown. The statistical analysis is described in Methods. *: *p* ˂ 0.05, *n* = 3. Whole Western blot figures are provided in Supplemental Materials Figure S6.

BCL2L10 was found to be overexpressed in several tumor types, but the mechanisms implicated in BCL2L10 expression have not been identified to date. Interestingly, analysis of microarray data generated from A375 melanoma cells treated with siRNAs against 45 transcription factors and signaling molecules (GSE31534) [31] revealed that the silencing of STAT3 markedly reduced BCL2L10 mRNA levels (Figure 3A). Furthermore, BCL2L10 mRNA levels were also reduced in three additional microarray studies that inhibited STAT3 expression or activity (GSE64536, GSE63092, and GSE48124, Supplementary Materials Figure S4). In line with these findings, we observed that in four out of the six patient samples tested, P-STAT3 staining overlapped with BCL2L10 staining. Unlike BCL2L10, which presented a heterogeneous pattern including both nuclear and cytoplasmic localization, P-STAT3 staining was almost exclusively nuclear in the melanoma samples (Supplementary Materials Figure S5).

The results above motivated us to determine whether STAT3 regulates BCL2L10 transcription. To perform this study, we used the cell lines UACC903 and WM9 since they have high levels of STAT3 phosphorylation at Tyr^705^, a marker of STAT3 activation (Figure 3B), together with an abundant BCL2L10 expression as showed in Figure 1A. Both cell lines were transduced with a scramble sequence, or two STAT3 specific pRetroSuper-based shRNAs (short-hairpin RNA) labeled shSTAT3-I and -II that efficiently knockdown STAT3 protein levels (Figure 3C). Importantly, the two STAT3 shRNA significantly reduced BCL2L10 protein levels in both cell lines, as demonstrated by the quantification of Western blots from three independent biological replicates (Figure 3C). Real-time PCR experiments revealed that STAT3 silencing in both WM9 and UACC903 cells led to a significant decrease in BCL2L10 mRNA levels (Figure 3D,E). These results indicate that STAT3 regulates BCL2L10 expression, most likely through the transactivation of its promoter.

### 2.3. STAT3 Binds the Human BCL2L10 Promoter and Activates BCL2L10 Transcription

**Figure 4:**
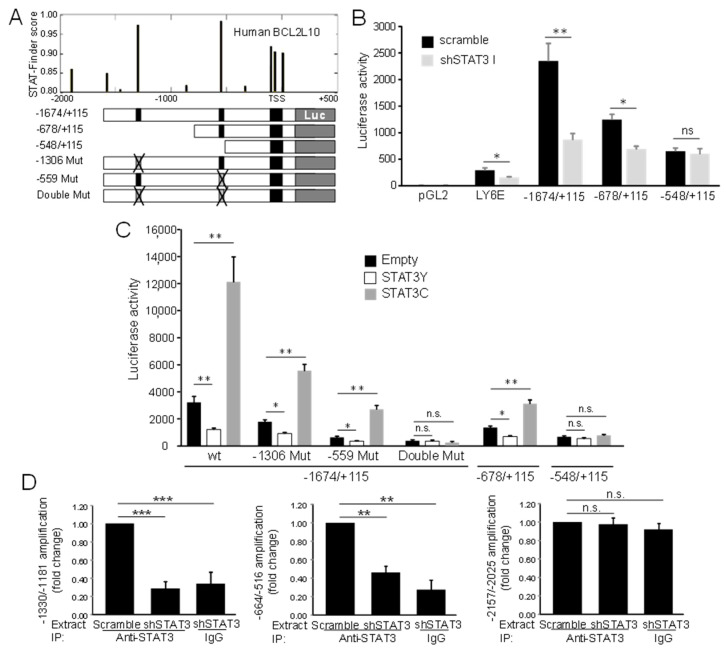
STAT3 binds to the BCL2L10 promoter and regulates its transcription. (**A**) Structure of the proximal region of the human BCL2L10 promoter. Putative STAT3 responsive elements (above) and fragments of the promoter that were cloned into pGL2 (below) are depicted. The sites at −1306 and −559 were deleted by site-directed mutagenesis, generating two plasmids with a single mutation and one plasmid with a double mutation. (**B**) Silencing of STAT3 diminished the reporter activity of the BCL2L10 promoter. The indicated reporter plasmids were transfected into UACC903-shSTAT3 and UACC903-scramble cells. Results are shown as the mean ± SD *: *p* ˂ 0.05. **: *p* ˂ 0.01, ns: not significant, *n* = 3. (**C**) STAT3 enhances BCL2L10 transactivation through the SREs at −1306 and −559. The indicated reporter plasmids were transfected into HEK293T cells together with STAT3Y and STAT3C plasmids. Results are shown as the mean ± SD *: *p* ˂ 0.05, **: *p* ˂ 0.01, ns: not significant, *n* = 3. (**D**) STAT3 silencing decreased binding of STAT3 to the BCL2L10 promoter. The plots show the relative level of BCL2L10 amplification (normalized to GAPDH levels) corresponding to region −1330/−1181 (left) and region −664/−516 (center) following a chromatin immunoprecipitation assay on UACC903-scramble and UACC903-shSTAT3 cells. Amplification of region −2157/−2025, used as a negative control, is shown on the right. The fold change compared to the level of amplification observed in UACC903-scramble cells is shown. The mean ± SD from three independent experiments is shown. The statistical analysis is described in Methods. **: *p* < 0.01 ***: *p* < 0.001, *n* = 3. n.s. = not significant.

To study the regulation of BCL2L10 transcription by STAT3, bioinformatic analysis of the human BCL2L10 gene was performed at Dr. Joo-Yeon Yoo’s laboratory. Analysis of the BCL2L10 promoter region (from −2.0 kb to +0.5 kb) using the STAT3-Finder software [32] revealed ten putative STAT3 responsive elements (SRE) with scores exceeding the cutoff value (>0.8, Figure 4A). The site at −559 was the only one conserved among mammals (data not shown). The sites at positions −559 (TTCTCAGAA) and −1306 (CCCCCAGAA) of the BCL2L10 promoter showed a score of 0.97 and 0.98, respectively and hence were considered to be the most likely functional SRE in the human BCL2L10 gene. Thus, the region between −1674 to +115 of the human BCL2L10 promoter that contains both candidate SREs and a cluster of SREs close to the transcription start was cloned into a luciferase reporter plasmid. Thereafter, we generated two deletion constructs that contained one (plasmid −678/+115) and none (plasmid −548/+115) of the two high ranked sites (Figure 4A). When transfected into UACC903 control cells, the three promoter fragments (−1674/+115, −678/+115 and −548/+115) displayed a high degree of activity (more than 200 times) compared with a promotorless pGL2 vector used as a control (data not shown). Then, we compared the reporter activity of these fragments in both UACC903-scramble and UACC903-shSTAT3 cells. As expected, the activity of Ly6E, a STAT reporter, is inhibited in UACC903-shSTAT3 compared with UACC903-scramble cells (Figure 4B). The activity elicited by constructs −1674/+115 and −678/+115 significantly decreased when transfected into UACC903-shSTAT3 cells compared to that observed when transfecting UACC903-scramble cells (Figure 4B).

The reduction seen upon STAT3 silencing was greater for plasmid −1674/+ 115 (70% of reduction) than for plasmid −678/+115 (43% of reduction). This result is consistent with the presence of two and one SREs in plasmids −1674/+115 and −678/+115, respectively. On the contrary, the shortest promoter fragment (−548/+115) showed similar luciferase activity both in UACC903-shSTAT3 and UACC903-scramble cells, indicating that the putative SREs in the cluster close to the transcription start are not functional. These results suggest that STAT3 regulates BCL2L10 transcription through responsive elements present in the region encompassed between nucleotides −1674 and −548. To confirm this result, we evaluated the luciferase activity in HEK293T cells cotransfected with plasmids encoding STAT3Y>F, a STAT3 dominant-negative mutant, STAT3C, a constitutively active STAT3 mutant, or empty plasmid. Expression of STAT3Y>F closely reproduced the reduction on BCL2L10 transcriptional activity seen in −1674/+115 and −678/+115 plasmids upon STAT3 silencing (Figure 4C). Meanwhile, transfection with STAT3C significantly increased the transcriptional activity driven by these two promoter constructs (−1674/+115 and −678/+115) over the level observed with empty plasmid (Figure 4C). To determine whether STAT3 mediates transcription of the BCL2L10 promoter through the putative SREs located at −1306 and −559, we generated two reporter plasmids with mutations that destroyed each of these sites (−1306 Mut and −559 Mut, displayed in Figure 3A). The mutation of either of these sites reduced the reporter activity compared to that observed with the wt BCL2L10 promoter (Figure 4C). Moreover, transfection with STAT3Y > F further reduced the activity of both promoter fragments indicating that the mutant reporters are still transactivated by STAT3. In line with this data, STAT3C increased the activity of both mutant promoters. In contrast, the plasmid with simultaneous mutation of both sites (double mut plasmid) presented not only a low luciferase activity but also a similar reporter activity in cells transfected with empty plasmid and the STAT3 mutants, indicating that this promoter fragment had completely lost STAT3 responsiveness (Figure 4C). Further support for the role of STAT3 in the regulation of BCL2L10 transcription comes from ChIP analysis. To this end, sheared chromatin was immunoprecipitated with antibodies to STAT3 (or control IgG) followed by real-time PCR amplification of BCL2L10 promoter sequences bearing putative SRE. Immunoprecipitation of STAT3 enabled specific amplification of DNA fragments corresponding to regions −1330 to −1181 (Figure 4D, above) and −664 to −516 (Figure 4D, below), but not of the fragment −2157 to −2025 that served as a negative control. The silencing of STAT3 reduced the amount of DNA amplified from the chromatin after STAT3 immunoprecipitation to values similar to those observed after immunoprecipitation with control IgG (Figure 4D). Altogether, these results indicate that STAT3 transactivates BCL2L10 through two SREs located at −1306 and −559 that display the classical STAT3 sequences CCCCCAGAA and TTCTCAGAA, respectively.

### 2.4. BCL2L10 Contributes to Melanoma Cell Survival

#### 2.4.1. BCL2L10 Enhances the Survival of Melanoma Cells Treated with DNA-Damaging Agents

**Figure 5:**
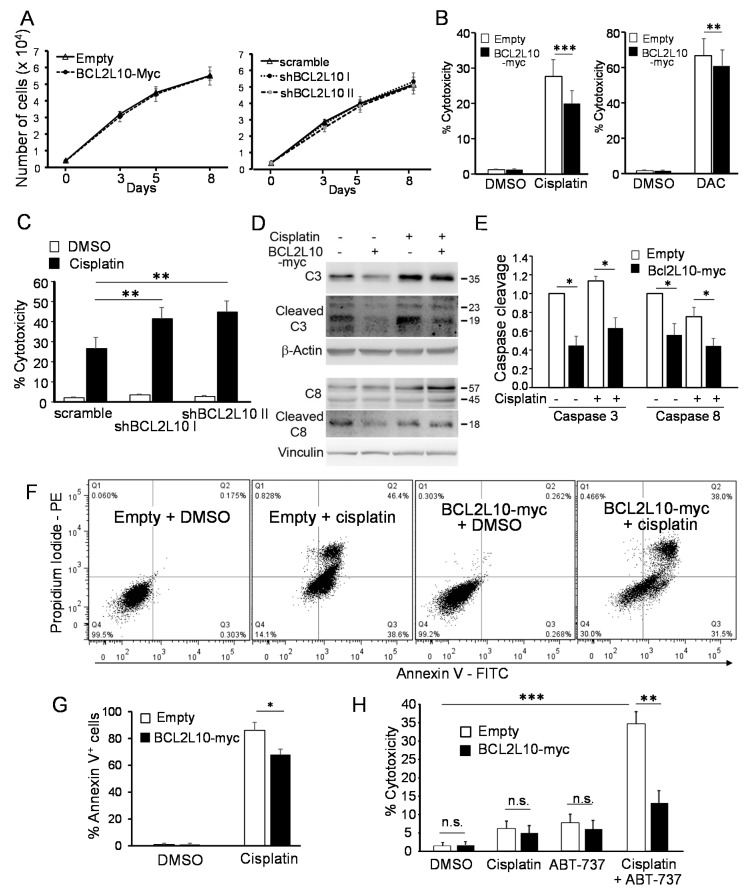
BCL2L10 enhances cell survival in melanoma upon treatment with DNA-damaging agents. (**A**) BCL2L10 does not affect cell growth. M2-empty and M2-BCL2L10-myc (left) and A375-scramble, A375-shBCL2L10 I, and A375-shBCL2L10 II cell lines (right) were grown for 8 days and the cell density was determined using a crystal violet assay. Results are shown as the mean ± SD, *n* = 3. (**B**) M2-empty and M2-BCL2L10-myc were treated with 40 μM cisplatin (left) or 100 μg/mL dacarbazine (DAC, right) for 48 h. Bar graph shows the mean ± SD (*n* = 7 for cisplatin and *n* = 5 for dacarbazine) of the percent of cytotoxicity. The percentage of cytotoxicity was calculated as the quotient between the number of cells in treated wells and the number of cells in non-treated wells times 100. (**C**) A375-scramble, A375-shBCL2L10 I, and A375-shBCL2L10 II cells were treated with cisplatin (40 μM) for 48 h. Bar graph shows the mean ± SD (from three independent experiments) of the percent of cytotoxicity. (**D**) M2-empty and M2-BCL2L10-myc were treated with 40 μM cisplatin for 24 h. Protein extracts were probed with antibodies against full-length caspase 3 (C3), cleaved caspase 3 (cleaved C3) or caspase 8 (C8). β-actin and vinculin were used as loading control. The blots displayed are representative of three independent experiments. (**E**) Bar graphs show the ratio (mean ± SD) between cleaved (19 kDa fragment for caspase 3 and 18 kDa fragment for caspase 8) and uncleaved full-length caspase 3 or caspase 8 from the experiment in (**D**). This ratio is expressed as the fold change relative to the ratio measured in DMSO-treated M2-empty cells. *: *p* < 0.05, *n* = 3. (**F**) M2-empty and M2-BCL2L10-myc were treated with 40 μM cisplatin for 24 h, stained with PI/annexin V and analyzed by flow cytometry. Representative histograms are shown. (**G**) Bar graph shows the mean ± SD (*n* = 3) of the percentage of annexin V positive cells from the experiment in (**F**). * *p* < 0.05, *n* = 3. (**H**) M2-empty and M2-BCL2L10-myc cells were treated with ABT-737 (2.5 μM), cisplatin (10 μM) or ABT-737 (2.5 μM) plus cisplatin (10 μM). Bar graph shows the mean ± SD (from three independent experiments) of the percent of cytotoxicity. The statistical analysis is described in Methods. **: *p* < 0.01, ***: *p* < 0.001, ns: not significant, *n* = 3. Whole Western blot figures are provided in Supplemental Materials Figure S7.

To investigate the role of BCL2L10 in cell proliferation and survival, both gain- and loss-of-function approaches were employed. For the gain of function strategy, we used the M2 cells stably transfected with BCL2L10-myc already described in Figure 1C. The M2 cell line was selected since it presented the lowest endogenous BCL2L10 levels among our cell lines (Figure 1A). It is important to note that the level of exogenous BCL2L10 expressed in this cell line was similar to that of the endogenous protein (Figure 1C), and therefore, the augmentation on BCL2L10 level was within a physiological range. For the loss of function approach, we silenced BCL2L10 expression by shRNA in A375 (Figure 1D), one of the cell lines that expressed the highest levels of endogenous BCL2L10 among the melanoma cell lines tested (Figure 1A). Since BCL2L10 was shown to regulate cell proliferation in both ovarian and hepatocellular carcinoma [15,23], we first evaluated the impact of altering BCL2L10 level on melanoma cell growth. We determined that neither BCL2L10 overexpression nor silencing affected cell growth (Figure 5A).

Next, we sought to evaluate changes in the cellular response to standard chemotherapeutic agents. For these experiments, we used a crystal violet cytotoxicity assay that, in agreement with previous publications [33,34,35,36], proved in our hands to be more reliable and sensitive than MTT and other methods to examine the impact of cytotoxic drugs on cell survival. Overexpression of BCL2L10-myc significantly reduced the cytotoxicity induced by cisplatin and dacarbazine (Figure 5B) in comparison to M2-empty cells. Similarly, the silencing of BCL2L10 sensitized A375 cells to cisplatin in comparison with A375-scramble cells (Figure 5C). To confirm these results, we analyzed cell death by both annexin V/PI staining and caspase cleavage in M2-empty and M2-BCL2L10-myc cells treated with cisplatin. Overexpression of BCL2L10-myc reduced both caspase 3 and caspase 8 cleavage, measured as the ratio of cleaved over uncleaved forms (Figure 5D,E). Further, BCL2L10-myc reduced the amount of annexin V positive cells upon cisplatin treatment (Figure 5F,G). These results indicate that BCL2L10 promotes cell survival in cells subjected to treatment with DNA-damaging agents.

Since Bcl-2 proteins are in many cases functionally redundant [37,38,39], we reasoned that the pro-survival activity of BCL2L10 in these experiments might have been curtailed by the expression of other anti-apoptotic Bcl-2 family members with a similar role. To address this question, we evaluated the effect of BCL2L10 expression in cisplatin-treated cells in which the contribution of other anti-apoptotic Bcl-2 proteins was neutralized by the addition of ABT-737 [40]. This compound is a BH3 mimetic that binds with high affinity to BCL2, Bcl-xL, and Bcl-w but does not inhibit MCL1, Bfl-1, and BCL2L10 [40]. Since the combination of ABT-737 and cisplatin has a synergistic killing effect in melanoma cells [41], we reduced the concentration of cisplatin from 40 to 10 μM to avoid excessive cell death. The treatment with 10 μM cisplatin or 2.5 μM ABT-737 induced less than 10% of cell death in M2-empty cells but adding both compounds together increased the cytotoxicity up to 34.7% in these cells (Figure 5H). However, the expression of BCL2L10 significantly reduced the cytotoxicity induced by the combination treatment to 13.7% (Figure 5H). This result confirms that BCL2L10 contributes to cisplatin resistance.

#### 2.4.2. BCL2L10 Promotes Resistance to ABT-737

**Figure 6:**
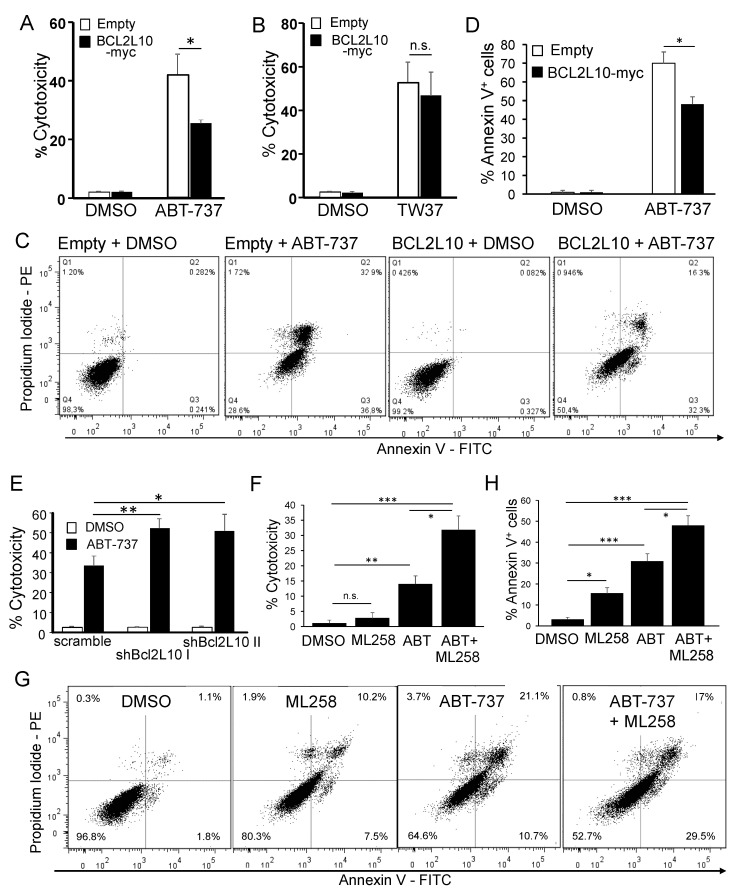
BCL2L10 promotes resistance to ABT-737. (**A**,**B**) M2-empty and M2-BCL2L10-myc cells were treated with 10 μM ABT-737 (**A**) or 5 μM TW-37 (**B**) for 48 h. Bar graph shows the mean ± SD (*n* = 4 for cisplatin and *n* = 5 for TW-37) of the percent of cytotoxicity. (**C**) M2-empty and M2-BCL2L10-myc cells were treated with 10 μM ABT-737 for 24 h, stained with PI/annexin V and analyzed by flow cytometry. Representative histograms are shown. (**D**) Bar graph shows the mean ± SD (*n* = 3) of the percentage of annexin V positive cells from the experiment in (**C**). * *p* < 0.01. ** *p* < 0.001, ns: not significant, *n* = 3. (**E**) A375-scramble and A375-shBCL2L10 cells were treated with ABT-737 (10 μM). Bar graph shows the mean ± SD (from three independent experiments) of the percent of cytotoxicity. (**F**) A375 cells were treated with ABT-737 (2.5 μM), ML258 (10 μM) or ABT-737 plus ML258 (same concentrations) and the percent of cytotoxicity was determined by using a crystal violet cytotoxicity assay. Bar graph shows the mean ± SD (from three independent experiments) of the percent of cytotoxicity. *: *p* < 0.05, *n* = 3. (**F**) A375 cells were treated with ABT-737 (2.5 μM), ML258 (10 μM) (alone or in combination) Bar graph shows the mean ± SD (*n* = 3) of the percent of cytotoxicity. (**G**) A375 cells were treated with ABT-737 (10 μM), ML258 (40 μM) (alone or in combination) for 24 h, stained with PI/annexin V and analyzed by flow cytometry. Representative histograms are shown. (**H**) Bar graph shows the mean ± SD (*n* = 3) of the percentage of annexin V positive cells from the experiment in (**G**). The statistical analysis is described in the Methods. *: *p* < 0.05, **: *p* < 0.01, ***: *p* < 0.001, ns: not significant, *n* = 3.

Since BCL2L10 (our results above), BCL2 [42,43], Bcl-xL [44], and MCL1 [45] have all been implicated in cisplatin resistance in melanoma and the partial protection to cisplatin provided by BCL2L10 was much greater in the presence of ABT-737 (34.7% vs. 13.7%, Figure 5H) than in its absence (27.6% vs. 19.7%, Figure 5B), it can be concluded that, at least in regard to cisplatin resistance, BCL2L10 can take over functions of the ABT-737 targets when they are inhibited by this drug. This conclusion evidences there are functional similarities between BCL2L10 and ABT-737 targets. To explore this in further depth without the confounding effect of cisplatin-induced cytotoxicity, we sought to assess the cell death induced exclusively by ABT-737 or TW-37 [46], a BH3-mimetic that targets MCL1 primarily. Although ABT-737 is usually poorly cytotoxic in melanoma (Figure 5H), it has been shown that higher doses can induce a modest, “on-target”, cell death [47]. Increasing ABT-737 to 10 μM induced 41.5% of cytotoxicity in M2-Empty cells, but significantly less (25.2%) in M2-BCL2L10-myc cells (Figure 6A). In contrast, the cytotoxicity induced by TW-37 was not affected by BCL2L10 expression (Figure 6B). Similarly, BCL2L10-myc reduced the amount of annexin V positive cells (Figure 6C,D) following ABT-737 treatment. These results suggest that BCL2L10 mediates resistance to ABT-737. This antagonistic effect of BCL2L10 and ABT-737 reinforces the notion that BCL2L10 can play a similar role than the ABT-737 targets BCL2, Bcl-xL or Bcl-w.

Next, we studied this matter in BCL2L10-knockdown cells. We observed that ABT-737 induced greater cytotoxicity in A375-shBCL2L10 cells compared to A375-scramble cells (Figure 6E), confirming that BCL2L10 is implicated in the resistance of melanoma cells to ABT-737.

Since the concentration used of ABT-737 is considered a high one, it is improbable that this effect is due to a sub-optimal inhibition of Bcl-2 proteins by the drug. Instead, the additional cell death observed in A375-shBCL2L10 cells can be attributed to functions of BCL2L10 not fulfilled by the ABT-737 targets. To confirm this result by using an alternative approach to suppress the BCL2L10 function, we used the compound ML258. ML258 is a BH3 mimetic t as a highly specific inhibitor of the Bim:BCL2L10 interaction [41]. ML258 had a poor cytotoxic effect by itself but induced a significant increase in cell death in the presence of ABT-737 as determined by both crystal violet cytotoxicity assay (Figure 6F) and annexin V staining (Figure 6G,H), confirming the results obtained in cells with knockdown of BCL2L10. Altogether, these results indicate that BCL2L10 is implicated in ABT-737 resistance, and it has both shared and distinctive functions when compared with ABT-737 targets, BCL2, Bcl-xL, and Bcl-w.

#### 2.4.3. BCL2L10 Enhances the Survival of Melanoma Cells Treated with a Combination of PLX-4032 and cisplatin or ABT-737

**Figure 7:**
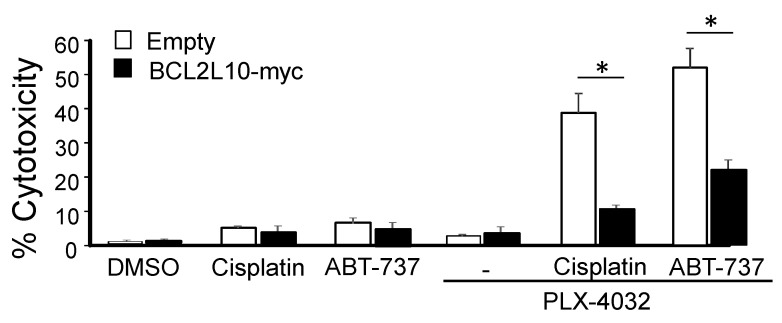
BCL2L10 protects melanoma cells from cell death upon treatment with PLX-4032 plus cisplatin or ABT-737. M2-empty and M2-BCL2L10-myc were treated for 48 h with the indicated drugs at the following concentrations: PLX-4032, 1 μM; cisplatin, 10 μM; ABT-737, 2.5 μM. The percent of cytotoxicity was determined by using a crystal violet cytotoxicity assay. Bar graph shows the mean ± SD (from three independent experiments) of the percent of cytotoxicity. The statistical analysis is described in Methods. *: *p* < 0.05.

BRAF inhibition is one of the current approaches to treat melanoma patients. Since targeting BRAF in melanoma patients proved unsuccessful in the long term, the combined use of BRAF inhibitors with other therapeutic strategies (i.e., immunotherapy, standard chemotherapy or BH3-mimetics) is currently being studied. Following our finding that BCL2L10 is implicated in melanoma resistance to cisplatin and ABT-737, we wanted to evaluate whether the effect of BCL2L10 expression in response to these drugs was also observed in the context of simultaneous inhibition of BRAF by the BRAF^V600E^ inhibitor PLX-4032. As expected, both combination treatments had greater cytotoxicity than either drug alone. We found that M2-BCL2L10-myc cells presented a significantly reduced cytotoxicity compared with M2-empty cells upon treatment with both PLX-4032 plus cisplatin and PLX-4032 plus ABT-737 (Figure 7). This result indicates that BCL2L10 expression protects melanoma cells from cisplatin and ABT-737, even when these drugs are combined with BRAF inhibitors.

## 3. Discussion

Mitochondrial-mediated apoptosis is regulated by a delicate balance of the opposing actions of pro-apoptotic and anti-apoptotic Bcl-2 family members. Therefore, the life/death decision is determined by the relative abundance of the Bcl-2 proteins on either “side” and their binding profiles [10]. Thus, the anti-apoptotic Bcl-2 proteins represent an interesting target for cancer therapy since its inhibition will unleash the pro-apoptotic function. This goal has been achieved using small molecule inhibitors generically named BH3-mimetics that compete with BH3-only proteins for the same hydrophobic groove on the anti-apoptotic proteins. As a result, the pro-apoptotic proteins are no longer inhibited and can promote the release and activation of Bax and/or Bak, the executors of MOMP [9,10]. Since different BH3-mimetic drugs target different anti-apoptotic Bcl-2 proteins and the Bcl-2 proteins are known to be functionally redundant [37,38,39], any rational attempt to inhibit these proteins must ideally begin by understanding which of the anti-apoptotic Bcl-2 proteins are expressed in a given tumor type, in a given patient, and under which circumstances. Otherwise, these therapeutic attempts are bound to fail, as happened with the therapies targeting BCL2 or BCL2/Bcl-xL in tumors rich in MCL1 [48,49,50,51]. The surveying of anti-apoptotic Bcl-2 proteins in different tumor types led to a focus on the members more frequently expressed: BCL2, Bcl-xL, and MCL1 [11,52]. In contrast, our knowledge of the function of the other three members of the family (Bcl-w, Bfl-1, and BCL2L10) has lagged behind. The study of Bcl-2 proteins in melanoma followed the same trend and also focused on BCL2, Bcl-xL, and MCL1 [53,54] although, to our knowledge, a comprehensive study of the relative expression of all six anti-apoptotic Bcl-2 proteins in melanoma cells or tumors has not been performed yet. In the present study, we have determined that BCL2L10 is expressed both frequently and at elevated levels in both melanoma cell lines and tissues from melanoma patients. One limitation of our study is that the reduced size of our patient cohort prevents us from analyzing changes in BCL2L10 expression in different stages of melanoma. It is important to mention that Placzek et al. in 2010 used quantitative PCR techniques to evaluate the mRNA expression levels of all six anti-apoptotic Bcl-2 subfamily members in 69 cancer cell lines, including 12 melanoma cell lines [55]. The expression of BCL2L10 in this study was disparaged since all except one cell line (68 out of the 69, including all 12 from melanoma) presented BCL2L10 levels below the baseline. The absence of BCL2L10 expression in cell lines from leukemia and from breast, prostate, colorectal, and lung cancers in this study is difficult to reconcile with the later observation that BCL2L10 is overexpressed in all these tumor types [27,28,29,30]. Moreover, four of the 12 melanoma cell lines were included in our study, and they all showed strong expression of BCL2L10 as assessed by Western blot. This important discrepancy most likely reflects the inaccuracy of profiling BCL2L10 in cancer at the mRNA levels, as was remarked in recent publications [28,56].

Despite the fact that BCL2L10 was found to be overexpressed in several tumor types, the mechanisms underlying BCL2L10 overexpression have not been investigated. We have found that BCL2L10 expression is enhanced by STAT3, a transcription factor that has a critical role in the development and progression of human tumors by promoting uncontrolled cell proliferation and growth, cell survival, induction of angiogenesis, and the suppression of host immune surveillance [57]. STAT3 is a critical point of convergence downstream of several hyperactive tyrosine kinase receptors that are either mutated or amplified in melanoma, such as KIT, ERBB4, EPH, FGFR, EGFR and PDGRFA, among others [58]. Accordingly, persistent phosphorylation of STAT3 at Tyr^705^ and elevated STAT3-dependent transactivation of target genes has been documented and implicated in melanoma progression [59,60]. Here, we have identified two STAT3 responsive elements in the BCL2L10 promoter that are responsible for strong STAT3-dependent BCL2L10 transcription. Interestingly, STAT3 has been implicated in cancer cell survival by regulating the transcription of other anti-apoptotic members of the Bcl-2 family, such as BCL2, Bcl-xL, and MCL1 [61,62,63]. Since we observed BCL2L10 transactivation by STAT3 in the model cell line HEK293, it is very likely that STAT3 drives BCL2L10 upregulation in other tumor types presenting constitutive activity of STAT3.

Considering that anti-apoptotic Bcl-2 proteins were and are evaluated as prospective therapeutic targets in melanoma [64], it is surprising that the role of BCL2L10 in melanoma has never been studied. Since BCL2L10 was shown to both promote and inhibit cell death in different systems, to elucidate the role of BCL2L10 in melanoma is not trivial. Our data established that BCL2L10 is a pro-survival protein that contributes to protecting melanoma cells from the DNA-damaging agents cisplatin and dacarbazine. In this regard, this function of BCL2L10 is similar to that of BCL2, Bcl-xL, and MCL1 since they are both abundantly expressed in melanoma and implicated in cisplatin resistance [42,43,44,45]. In addition, the three approaches we used through this work (BCL2L10 overexpression and BCL2L10 genetic and chemical inhibition) indicate that BCL2L10 is an important factor contributing to ABT-737 resistance. This observation is in agreement with the previous finding that BCL2L10 expression caused ABT-737 resistance in the acute lymphoblastic leukemia cell lines J16 and MOLT-4 [65]. Altogether, these findings position BCL2L10 as an important new pro-survival factor in melanoma.

A critical step to further understand BCL2L10′s role is to determine whether BCL2L10 has either distinctive or overlapping functions with the other five Bcl-2 anti-apoptotic proteins. Our data show that BCL2L10 expression inhibits cytotoxicity induced by ABT-737 but not by TW-37. This observation suggests that there is no overlap between MCL1 and BCL2L10 function. The most likely reason is that they partner with different BH3-only sensitizers proteins; Noxa and Hrk interact with MCL1 and BIK with BCL2L10. Another implication of the finding above is that BCL2L10 shares functions with ABT-737 targets. Similar to BCL2L10, Bcl-xL and Bcl-w also interact with Bik, apart from other sensitizers. Hence, it is tempting to speculate that these two proteins, rather than BCL2 (that interacts with Bmf and Bad, but not with Bik), are the ones that present some functional similitudes with BCL2L10. Therefore, the very restricted (Bik and Bim) and unique BH3-only protein binding profile of BCL2L10 may determine its biological function. Moreover, unlike the other five Bcl-2 anti-apoptotic proteins, BCL2L10 exclusively inhibits Bax-dependent cell death and is not involved in Bid or Bak-dependent apoptosis. These observations are consistent with sequence and phylogenetic analysis that suggest BCL2L10 is the most divergent Bcl-2 anti-apoptotic member [66]. On the other hand, when we inhibited BCL2L10 by either shRNA or the ML258 inhibitor, we observed increased cell death, suggesting that BCL2L10 has distinct functions from that of ABT-737 targets. Although this observation does not contradict the previous conclusion that BCL2L10 functions overlap with that of ABT-737 targets, it adds another layer of complexity to the BCL2L10 function. Notwithstanding, these experiments just represent an initial exploration of the similitudes and differences of BCL2L10 with the other five Bcl-2 anti-apoptotic proteins.

The mechanisms underlying the pro-survival activity of BCL2L10 in melanoma have not been explored in this work. The conventional interpretation is that BCL2L10 regulates apoptosis by interacting with BH3-only proteins and inhibiting the intrinsic pathway of apoptosis [12,20,22,67]. However, it was also demonstrated that BCL2L10 prevents apoptosis by BH3-independent mechanisms and through the binding of Apaf-1 [19,21,68], a critical component of the apoptosome implicated in the cleavage of Procaspase-9. The anti-apoptotic effect of BCL2L10 was also shown to be the consequence of the inhibition of mitophagy [69] or the release of Ca2+ from the endoplasmic reticulum [29]. Future studies will determine specifically which of these mechanisms are operating in melanoma cells.

The constitutive activation of the BRAF/MEK/ERK pathway is a hallmark of melanoma. Pharmacological inhibition of this pathway strongly enhances the expression or activity of almost all Bcl-2 pro-apoptotic proteins [70]. However, the initial effect of BRAF inhibitors is usually not cytotoxic but cytostatic (i.e., G1 cell arrest) since the accumulation of pro-apoptotic BH3-only proteins is neutralized by the abundant amounts of anti-apoptotic proteins usually expressed by melanoma cells. However, the induction of BH3-only proteins by BRAF inhibition efficiently primes tumor cells for apoptosis, allowing them to straightforwardly tilt the balance toward cell death by co-targeting the pro-survival Bcl-2 family members. In agreement with this model, it has been demonstrated that ABT-737 and other BH3 mimetics combine synergistically with MAPK inhibitors in the killing of BRAF mutant melanoma cells [71,72,73]. These observations have provided the rationale to use this combination in melanoma patients (ABT-263 [74] plus dabrafenib and trametinib, clinical trial NCT01989585) with the intent to delay the onset of resistance to BRAF inhibitors. This phenomenon of apoptotic priming by BRAF inhibitors can also enhance the cytotoxicity of standard chemotherapy, suggesting that this combination still needs to be further investigated [75,76,77,78]. We evaluated the role of BCL2L10 in these two possible therapeutic scenarios. Even though we have demonstrated that BCL2L10 can prevent cell death by both cisplatin and ABT-737, in the presence of PLX-4032, the protective role of BCL2L10 was much more pronounced (compare Figure 7 vs. Figure 5B for cisplatin and Figure 7 vs. Figure 6A for ABT-737). This difference may be due to the upregulation of Bik, one of the few BCL2L10 partners, following ERK pathway inhibition [79,80]. If this hypothesis holds true, it may indicate that inhibition of BCL2L10 can be envisioned as an additional way to enhance the clinical efficacy of BRAF inhibition.

Altogether, our results indicate that the elevated expression of BCL2L10 in melanoma contributes to cell survival upon treatment with different cytotoxic compounds, making BCL2L10 a promising target in the context of inhibiting anti-apoptotic Bcl-2 proteins in malignant melanoma treatment.

## 4. Materials and Methods

### 4.1. Cell Culture

Melanoma cell lines were kindly provided by Dr. Zeev Ronai (Sanford Burnham Prebys Medical Discovery Institute, San Diego, CA, USA) except for the M2 cell line that was provided by Dr. S. Alvarez (IMIBIO-SL) [81]. All cell lines were maintained in DMEM supplemented with 10% fetal bovine serum (FBS, Invitrogen, Carlsbad, CA, USA) 100 U/mL penicillin and 100 mg/mL streptomycin (Invitrogen, Carlsbad, CA, USA) at 37 °C and 5% CO_2_. Cells were transfected with calcium phosphate or by lipofectamine PLUS reagent (Invitrogen) following the manufacturer’s protocol. The cell lines are free of mycoplasma contamination and were authenticated as described [82].

### 4.2. Plasmids and Viral Constructs

The BCL2L10 expression plasmid incorporating the N-terminal Myc epitope tag has been previously described [83]. This vector was introduced into M2 cells, and transfected cells were selected with 100 ug/mL Hygromycin B (Gibco). The oligonucleotides targeting STAT3 (5′-GCAGCAGCTGAACAACATG-3′ and 5′-GATTGACCTAGAGACCCAC-3′) [82] and scramble (5′-GAAACTGCTGACCGTTAAT-3′) were cloned into the pRetroSuper vector. Silencing of BCL2L10 was performed using pLKO.1 shRNA clones TRCN0000033595 and TRCN0000033596 designed by The RNAi Consortium (TRC) and previously validated [84]. Viral particles were generated as described [85].

### 4.3. Real-Time PCR

Real-time PCR was performed as described [86]. Specific primers used for PCR were as follows: BCL2L10 forward 5′ GCCTTCATTTATCTCTGGACAC3′; BCL2L10 reverse, 5′AAGGTGCTTTCCCTCAGTTC3′, RNPII forward 5′GCTGTGTCTGCTTCTTCTG3′, RNPII reverse 5′CGAACTTGTTGTCCATCTCC3′ RNPII (RNA Polymerase II) served as an endogenous control. Reactions were run in triplicate. The target mRNA concentration of control cells, normalized to the level of RNPII mRNA, was set to 1.

### 4.4. Chromatin Immunoprecipitation (ChIP)

For ChIP analysis, UACC903 cells were fixed with 11% formaldehyde and sheared chromatin was immunoprecipitated with a STAT3 antibody (sc-482, Santa Cruz Biotechnology, Dallas, TX) or control IgG and subjected to real-time PCR. The following primers corresponding to the proximal region of the BCL2L10 promoter were used: BCL2L10 (−1330/−1181) forward 5′GGCCTAGTAGCAAGGCAGAA3′ and BCL2L10 (−1330/−1181) reverse 5′GGCCTAGTAGCAAGGCAGAA3′ and BCL2L10 (−664/−516) forward 5′CTAAGACAGCTGCCAAGTGC3′ and BCL2L10 (−664/−516) reverse 5′TCCATTCTGCATCAGTCTGG3′. The primers BCL2L10 (−2157/−2025) forward 5′CTTTGGAGGGAGAATTCCA3′ and BCL2L10 (−2157/−2025) reverse 5′CAGATGGACAGAATTACATGC3′ were used as a control.

### 4.5. Luciferase Assays

The BCL2L10 promoter was amplified from genomic DNA from HUVEC cells using the primers: −1674 (forward) 5′CTCTTTCATGTGGTACCAGCAC3′ and +234 (reverse) 5′TTACGGCAGATTCACCGGTC3′. The purified product was amplified in a semi-nested PCR using the same forward primer and the reverse primer +115 5′GGGAGCGCACTCGAGCTGTTG3′. The smaller fragments were generated using the +115 reverse primer and the following forward primers: −703 5′ACCCAGTCTATGGCATTCTGC3′, −678 5′ GCCGCCTGGTACCAGACTAAGAC 3′ and −548 5′ CCACTGCTGGTA CCATTCTGC 3′. The three promoter fragments were cloned into the Xho I y Kpn I sites of the pGL2-Basic plasmid. Site-directed mutagenesis of STAT3 sites was performed using the QuikChange II kit (Stratagene, San Diego, CA, USA) following the manufacturer’s protocol. Cell lysates were prepared from lipofectamine-transfected cells after 24 or 48 h. Luciferase activity was measured with the luciferase assay system (Promega, Madison, WI, USA) in a Berthold luminometer (Berthold Technologies, Bad Wildbad, Germany, Germany) and was normalized with β-galactosidase activity measured in the same sample. Results are shown as the mean (bar) ± SD

### 4.6. Proliferation Assays

Cells (5 × 10^3^/well) were plated in a 96-well plate (8 wells per time-point) and incubated for 72 h with DMEM 10% FBS. Cells were fixed (4% PFA), washed and stained with 0.1% crystal violet in 10% ethanol for 30 min. Then, the crystal violet solution was recovered, and plates were washed by immersion and dried at 37 °C. Crystal violet was dissolved in 10% Acetic Acid. The absorbance (optical density (OD)) was detected at 590 nm with a μQuant microplate reader (Biotek Instruments, Winooski, VT, USA). A plate fixed at 6 h was used as a control of seeding. A standard calibration curve was used to convert OD to the number of cells.

### 4.7. Immunohistochemistry

All the melanoma samples were from the Hospital Israelita archives, currently at the Universidad Maimonides. Samples were deparaffinized, rehydrated, and subjected to heat-induced epitope retrieval using citrate buffer (10 mM, pH 6). The endogenous peroxidase activity was quenched by placing them in methanol, 1.5% H_2_O_2_ for 30 min. After washing, the slides were blocked with blocking solution (PBS, 3% BFS) for 30 min at room temperature. The slides were incubated with a 1:300 dilution of the anti-BCL2L10 antibody (PA5-22190, Invitrogen) for 1 h at 30 °C in a humidified chamber. The signal was detected using VECTASTAIN^®^ Elite ABC Kit according to manufacturer protocol, followed by the staining with the DAB Substrate kit, Peroxidase (HRP), with nickel (Vector, Burlingame, CA, USA), as indicated by the kit. Sections were counterstained with hematoxylin and analyzed by a pathologist.

### 4.8. Crystal Violet Cytotoxicity Assay

Cells (5 × 10^3^/well) were plated in 96-well plates and incubated for 24 h with DMEM 10% FBS. Thereafter, the testing compounds were added to the plate (in quadruplicates) and left for 48 h. The concentration of the drugs is indicated in the corresponding figure legend. Cells were incubated with DMSO as a control. After removal of the medium, the plates were rinsed with 100 μL PBS/well, fixed and stained with 200 μL of 0.1% crystal violet in 10% ethanol for 30 min. Plates were rinsed 4 times in tap water and dried at 37 °C. Crystal violet was dissolved in 10% acetic acid. The absorbance (optical density (OD)) was detected at 590 nm with a μQuant microplate reader (Biotek Instruments). A plate fixed at 6 h was used as a control of seeding. A standard calibration curve was used to convert OD to the number of cells. The percentage of cytotoxicity was calculated as the quotient between the number of cells in treated wells and the number of cells in untreated wells times 100.

### 4.9. Quantification of Apoptotic Cell Death

The M2 cells were seeded on 6-well plates at a density of 1.25 × 10^5^ cells per well. The following day they were exposed to 40 μM cisplatin or 10 μM ABT-737 for 24 h. A375 cells were treated with 10 μM ABT-737 and 40 μM ML258, either alone or in combination for 24 h. Cells were washed twice with PBS and resuspended in 100 µL of annexin V binding buffer (pH 7.4) (BD Biosciences, Franklin Lakes, NJ, USA). Then, annexin V-Alexa Fluor 488 (BD Biosciences) was added and incubated for 15 min under dark conditions. Propidium iodide (0.1 µg/mL; Sigma-Aldrich; Merck KGaA, Darmstadt, Germany) was added just prior to signal acquisition. Cells were analyzed using a FACSAria flow cytometer (BD Biosciences, San Jose, CA) and analyzed with FACSDiva 7.6.1 software (BD Biosciences).

### 4.10. Western Blotting

For the Western blotting analysis, cell lysates were collected by the addition of lysis buffer supplemented with protease and phosphatase inhibitors for 10 min on ice [87]. The cell lysates were centrifuged at 13,000 rpm for 15 min at 4 °C, and the supernatants were collected and quantified using the Bradford method. Between 20 and 50 μg of proteins were diluted in 6× Lemmli buffer, boiled at 95 °C for 5 min, separated on 8–12% SDS–PAGE gels and then transferred to nitrocellulose membrane. The membranes were blocked with 5% milk in 0.05% Tween-PBS at room temperature for 1 h and then incubated with the primary antibodies at 4 °C overnight. The following antibodies were used: GAPDH (sc-25,778), STAT3 (sc-482), pSTAT3 (sc-8059), and caspase-3 (sc-7148) from Santa Cruz Biotechnologies, cleaved caspase-3 (9664) from Cell Signaling (Danvers, MA, USA) and caspase-8 (66231A) from BD Pharmingen. The primary antibodies anti-BCL2L10 were from Invitrogen (PA5-22190) and from Cell Signaling (CS #3869) and were both used at a 1:1000 dilution. Antibodies to β-actin (A5441) and α-tubulin (T9026) were from Sigma. The corresponding HRP-conjugated secondary antibodies: anti-mouse (GE NA931V), anti-rabbit (GE NA934) or anti-goat (sc-2020) were incubated for 1 h at room temperature. Immunoreactive bands were detected by an ECL system (Amersham Biosciences, Buckinghamshire, UK) using an image reader (ImageQuant 350, GE Healthcare, Chicago, IL, USA). Quantification of band intensities was performed using ImageJ (NIH). The intensity of each band was normalized to GAPDH or another housekeeping gene (i.e., Tubulin or actin) and the fold change (FC) relative to control cells was calculated. To draw a conclusion on a particular experiment, at least three biological (independent) replicates of paired samples were examined to calculate the mean and standard deviation. The log transformation of FC values was calculated to obtain a more symmetric distribution that better suits the normality assumptions of the subsequent statistical tests.

### 4.11. Statistics

Except when indicated, experiments were performed at least 3 times. Mean differences between groups were determined using either Student’s *t*-tests (Figure 4B and Figure 5A (M2 cells), Figure 5B,E,G,H, Figure 6A,B,D and Figure 7) or one-way ANOVAs followed by *post hoc* tests (Figure 1D, Figure 3C–F, Figure 4C,D, Figure 5A (A375 cells), Figure 5C and Figure 6E,F,H). Values of *p* < 0.05 were considered statistically significant. Statistical analyses were conducted using software from GraphPad Prism.

## 5. Conclusions

The data presented here allow us to conclude that BCL2L10 is frequently and abundantly expressed in melanoma. BCL2L10 plays a pro-survival role by contributing to the resistance of melanoma cells to DNA-damaging agents and ABT-737. These functions were also observed in the context of BRAF inhibition, indicating that targeting BCL2L10 may enhance the clinical efficacy of other therapies against melanoma.

## Data Availability

All the data is contained within the article or supplementary material.

## Supplementary Materials

The following are available online at https://www.mdpi.com/2072-6694/13/1/78/s1, Figure S1: Whole Western blot figures for Figure 1, Figure S2: Antibodies PA5-22190 and #3869 have the same pattern of reaction against BCL2L10. Figure S3: Expression of other Bcl-2 protein family members was not affected by BCL2L10 overexpression. Figure S4: Analysis of GSE64536, GSE63092, and GSE48124 demonstrated that STAT3 inhibition reduced BCL2L10 mRNA levels. Figure S5: Overlap of P-STAT3 and BCL2L10 staining in melanoma samples, Figure S6: Whole Western blot figures for Figure 3, Figure S7: Whole Western blot figures for Figure 5 Table S1: Immunohistochemical analysis of BCL2L10 staining in samples from melanoma patients.

## Abbreviations

STAT3	Signal transducer and activator of transcription 3
BCL2	B-cell lymphoma 2
BCL2L10	BCL2 Like 10
SRE	STAT3 responsive elements
GAPDH	Glyceraldehyde-3-phosphate dehydrogenase
PI3K	Phosphoinositide-3-kinase
shRNA	Short hairpin RNA
RNPII	RNA polymerase II
MOMP	Mitochondrial outer membrane permeability
IHC	Immunohistochemistry
