# Supplementary material for: BCL2L10 Is Overexpressed in Melanoma Downstream of STAT3 and Promotes Cisplatin and ABT-737 Resistance
Source: Cancers (Basel). 2020 Dec 30;13(1):78. doi: 10.3390/cancers13010078 (PMC7795116; doi:10.3390/cancers13010078)
Supplement: Supplementary file 1 [file cancers-13-00078-s001.pdf]

Figure 1A The lane's order is the same as in the manuscript

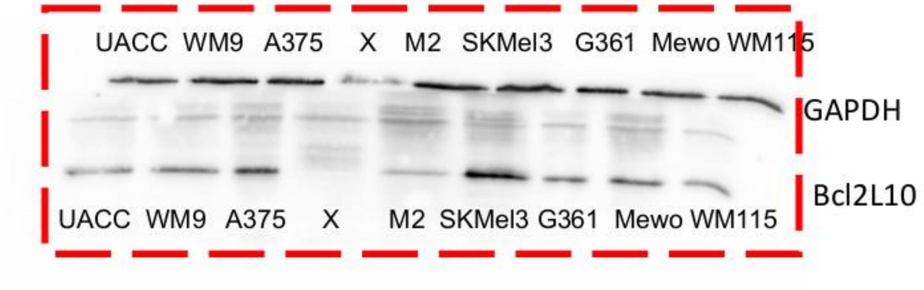

Lane X was discarded due to poor loading

Figure 1B Lanes 1 and 2

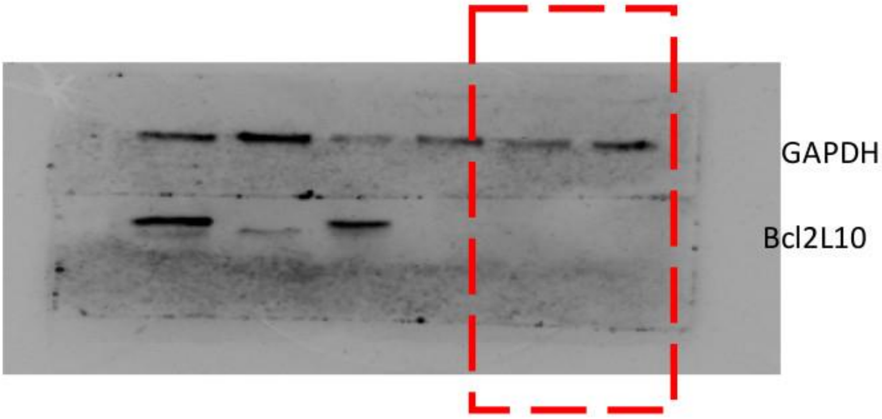

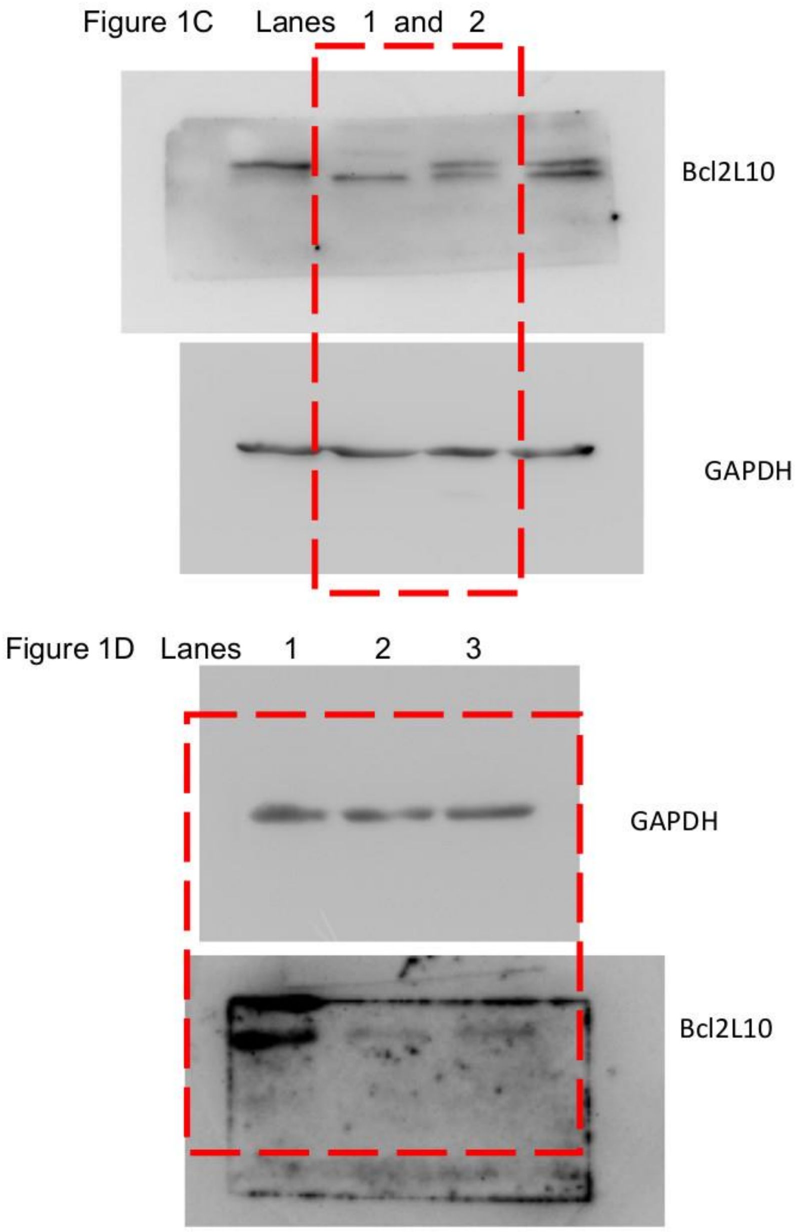

Figure S1. Whole Western blot figures for Figure 1.

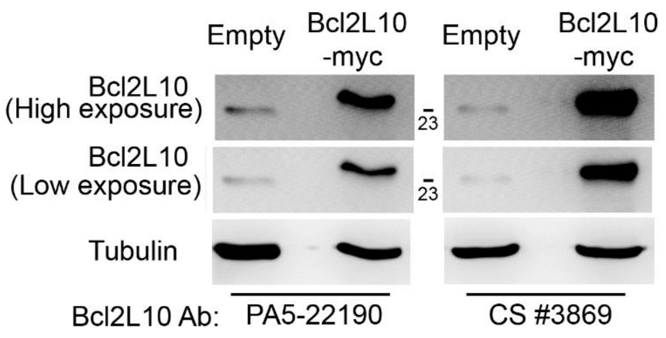

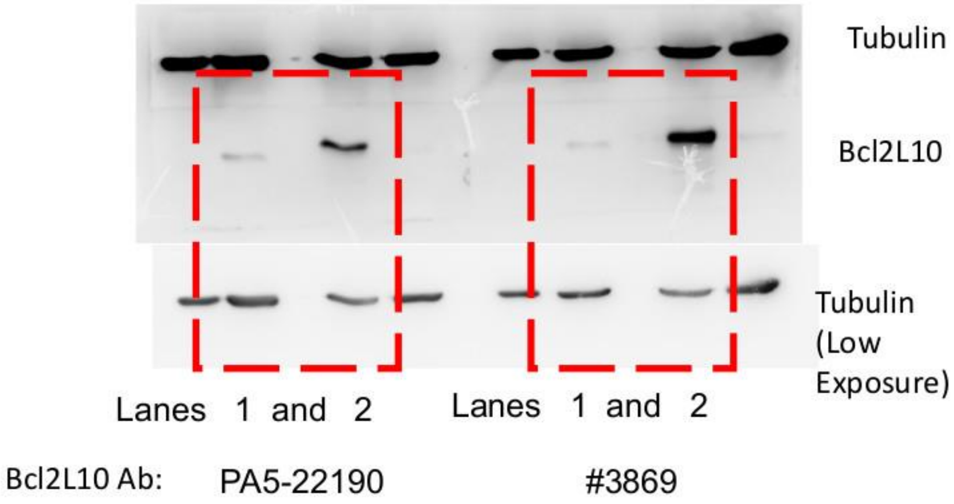

Figure S2. Antibodies PA5-22190 and #3869 have the same pattern of reaction against Bcl2L10.

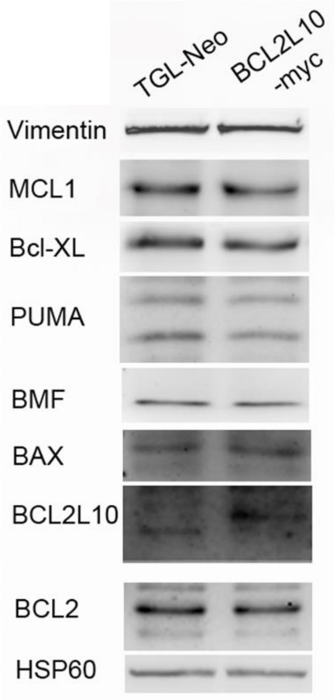

.

Figure 3. BCL2L10. overexpression in M2 cells does not affect the expression of proteins related with apoptosis. Vimentin and HSP60 were used as loading controls.

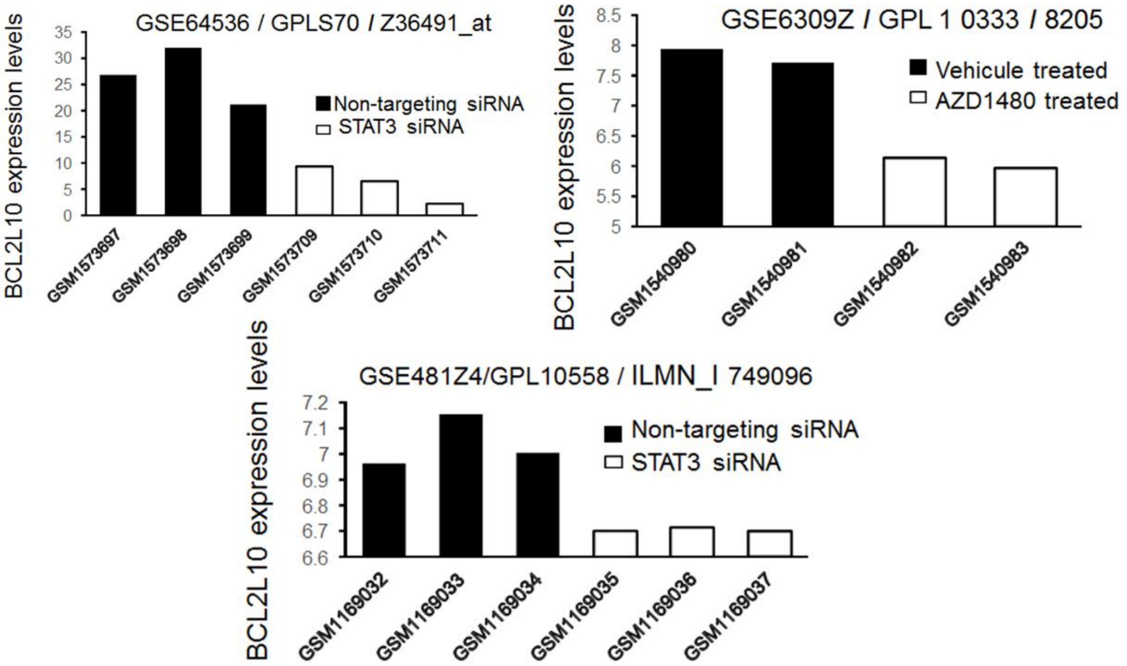

Figure S4. Analysis of GSE64536, GSE63092, and GSE48124 demonstrated that STAT3 inhibition reduced Bcl2L10 mRNA levels.

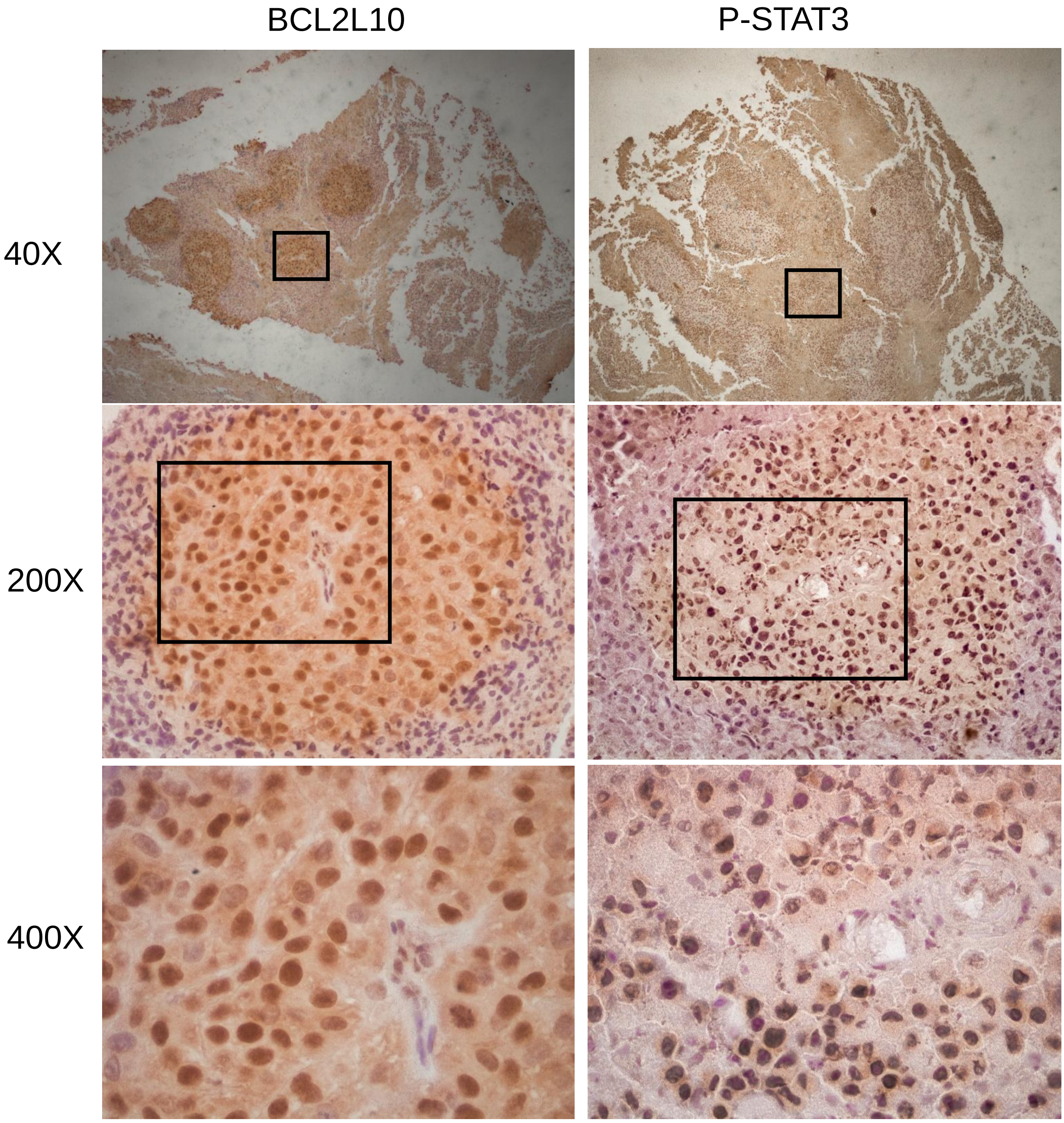

Figure S5. Representative images of immunohistochemistry staining of patient P110058 for BCL2L10 (PA5-22190 antibody, 1:300 dilution and P-STAT3 (sc-482 antibody, 1:100 dilution) with hematoxylin counterstaining. Nuclear P-STAT3 and nucleocytoplasmic BCL2L10 staining can be observed in the same areas of the tumor.

Figure 3B Lanes 1 to 4

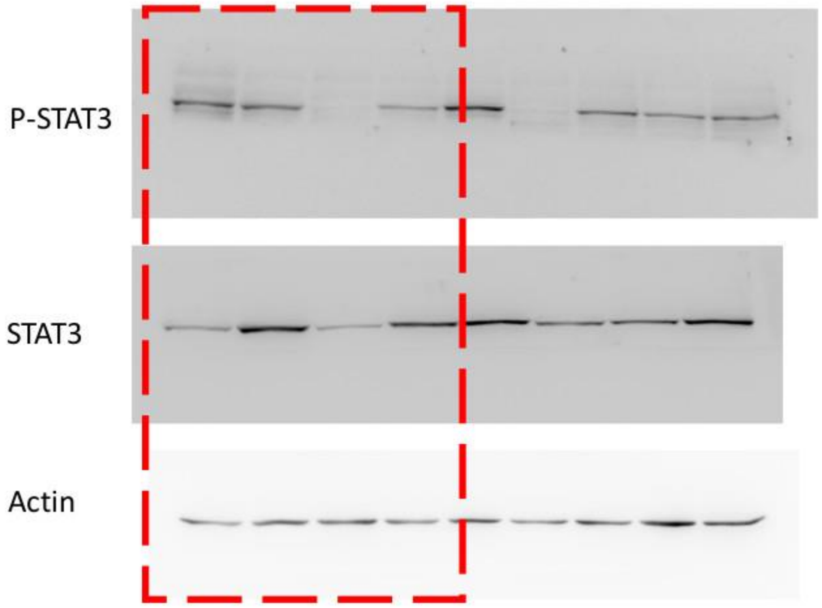

Figure 3C   Lanes 1   2   3   corresponding to WM9

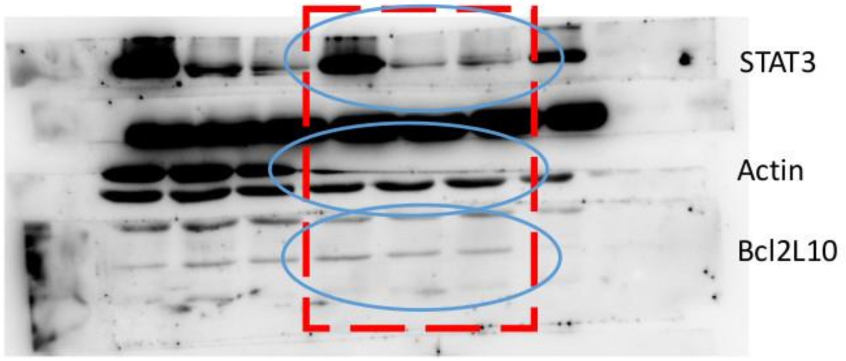

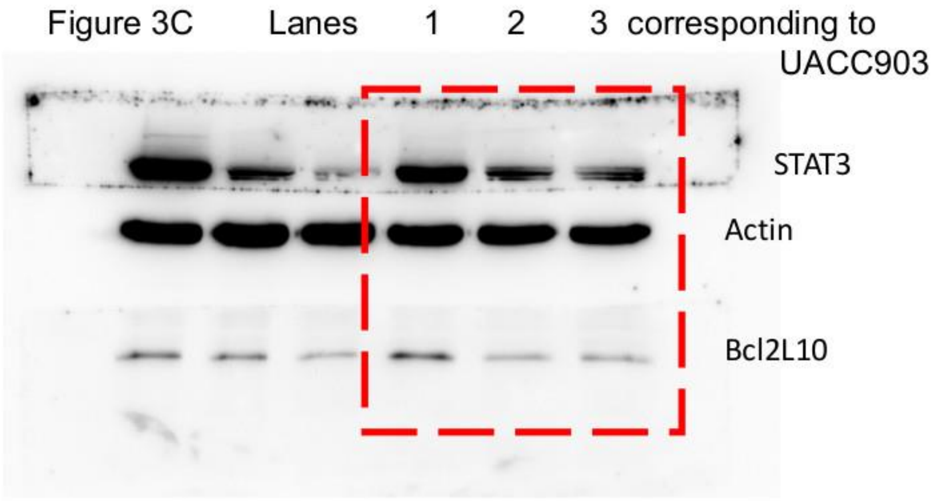

Figure S6. Whole Western blot figures for Figure 3.

Figure 5D

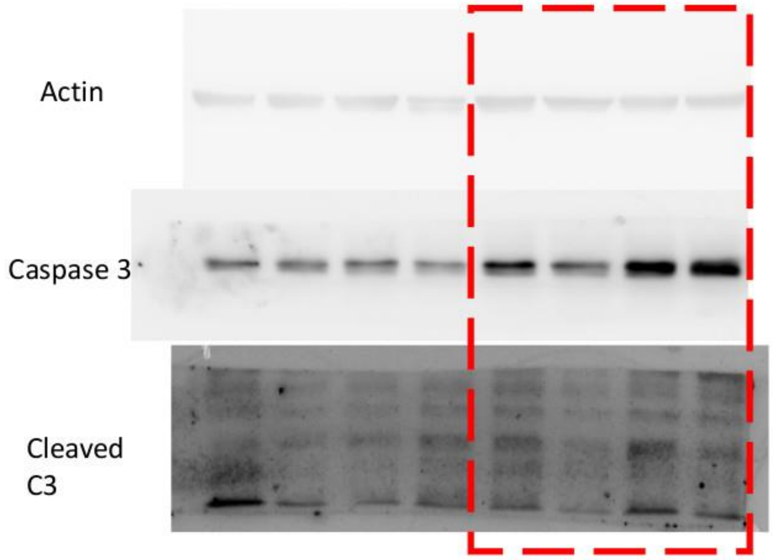

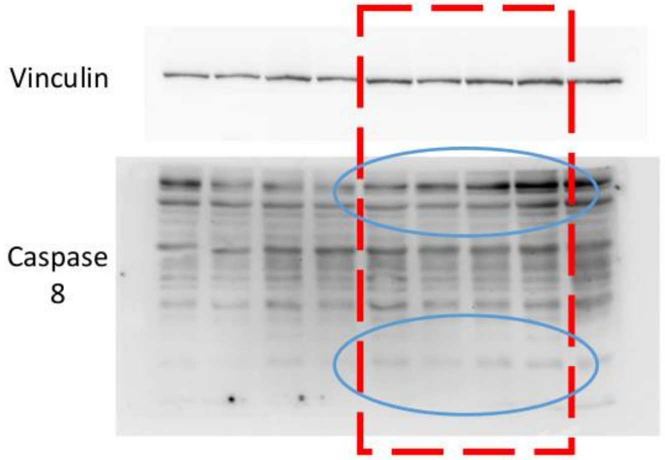

Figure S7. Whole Western blot figures for Figure 5.

Table S1. Immunohistochemical analysis of Bcl2L10 staining in melanoma tumor samples

Patient ID	Diagnosis	Clark	Bcl2L10 staining
117061 (1)	Skin melanoma	In situ	++
116871	Intradermic melanoma	I	++
84619	Nodular melanoma	II	+++
116235	Skin melanoma	II	+++
98125	Skin melanoma	III	-
97040 (1)	Skin melanoma	III	-
96700	Skin melanoma	III	++
104059	Skin melanoma	III	+++
103708	Skin melanoma	IV	++
99404	Skin melanoma	IV	++
98148	Skin melanoma	IV	++
104751	Skin melanoma	IV	+++
100205	Skin melanoma	IV	+++
97202	Skin melanoma	IV	+++
113369	Nodular melanoma	IV	+++
103279	Skin melanoma	-	++
113071	Lymph node metastasis		++
107251	Lymph node metastasis		+++
107366	Lymph node metastasis		+++
110058	Metastasis - Hypodermis		+++
